# Predictors of body appreciation in mothers of children living with ASD: the role of physical activity, self-compassion, and emotional eating

**DOI:** 10.3389/fnut.2026.1775100

**Published:** 2026-10-01

**Authors:** Marina Reuter Pierote, Isabela Pereira Rocon, Gabriela Grillo da Silva, Fabíola Lacerda Pires Soares

**Affiliations:** 1 Graduate Program in Nutrition and Health, Federal University of Espírito Santo, Vitória, Brazil; 2 Department of Nutrition, Federal University of Espírito Santo, Vitória, Brazil

**Keywords:** autism spectrum disorder, body appreciation, body image, emotional eating, mothers, self-compassion

## Abstract

**Introduction:**

Mothers of children living with Autism Spectrum Disorder (ASD) often experience high levels of emotional overload, which can negatively impact their eating behaviors and body image. In this context, body appreciation—a key facet of positive body image—serves as an important protective factor. However, evidence regarding the determinants of body appreciation among caregivers of children with ASD remains limited. This study aimed to investigate the sociodemographic, clinical, lifestyle, and psychological factors associated with body appreciation in mothers of children living with ASD.

**Materials and methods:**

This cross-sectional study evaluated 143 mothers of children with ASD residing in Espírito Santo, Brazil. Data were collected online through self-administered questionnaires assessing sociodemographic and clinical characteristics, nutritional status, lifestyle habits, self-compassion, emotional eating, and body appreciation. Multiple linear regression analysis was conducted using Jeffreys’s Amazing Statistics Program (JASP, version 0.18.0) to identify predictors of body appreciation.

**Results:**

Multiple regression analysis demonstrated that higher body appreciation was significantly predicted by engagement in physical activity (*β* = 0.293; *p* = 0.044), child’s age over six years (*β* = 0.329; *p* = 0.016), and higher self-compassion (*β* = 0.440; *p* < 0.001). High Body Mass Index (BMI) (*β* = -0.034; *p* = 0.005) and emotional eating (*β* = -0.010; *p* < 0.001) were inversely associated with body appreciation.

**Discussion:**

Self-compassion and physical activity emerged as key positive correlates of body appreciation, whereas elevated BMI and emotional eating were identified as negative predictors in this population. Given the cross-sectional design, longitudinal studies across diverse geographical settings are needed to confirm causality and support generalizability. Identifying these predictors provides actionable insights for designing targeted interventions aimed at promoting positive body image and mitigating dysfunctional eating behaviors in mothers of children with ASD.

## 1 Introduction

Autism Spectrum Disorder (ASD) is a complex neurodevelopmental condition in which genetic and non-genetic influences act either in isolation or in combination in the etiology of ASD (1). Symptoms usually appear in early childhood, although a formal diagnosis may occur later in life (2). The diagnosis of ASD reverberates beyond the individual on the spectrum, substantially impacting family dynamics (3). In this context, the treatment journey as well as the continuous care demands constitute chronic stressors for caregivers, often associated with overload, social isolation, and a scarcity of support networks (4). The challenges encountered in these parents’ daily lives increase concern and the self-blaming process regarding the diagnosis, influencing their mental health and coping mechanisms (5). In this scenario, literature demonstrates that the care burden falls disproportionately on maternal figures, due to their role as the child’s primary caregiver (6–8). Mothers of children living with ASD experience high levels of stress in their daily lives (6, 9) and suffer greater psychosocial impacts, since most of the time they are taking care of their child full-time. This emotional fragility, potentiated by the lack of social and marital support, makes these mothers vulnerable to negative health outcomes (9).

Among these outcomes, literature elucidates the close relationship between psychological stress and changes in eating behavior (10, 11), highlighting emotional eating as a coping strategy in the face of stressors (12–14). Such behavior emerges as a mechanism to deal with negative emotions, including stress (15). Furthermore, a positive association is noted between this practice and Body Mass Index (BMI), revealing that high levels of emotional eating are linked to higher weight (16), which is justified by the tendency of these individuals to consume hyper-palatable foods, rich in fat and sugar (17). In this sense, a cycle is established: in individuals with obesity, the desire for the thinness ideal intensifies stress and reduces body appreciation (18), a variable that shows a negative association with emotional eating (19).

Therefore, given the panorama in which these mothers are situated, it is crucial to evaluate how mental health impacts their eating behavior and relationship with body image. However, few studies have addressed these themes among caregivers of children diagnosed with ASD. Research by Radin et al. and Van Der Lubbe et al. has evidenced a positive association between stress and emotional eating in this population (8, 20). Furthermore, Shloim et al. highlight that maternal overload is frequently associated with a negative body image (21), a condition that can be potentiated in a stressful context such as the one faced by these caregivers. This body dissatisfaction may contribute to dysfunctional eating behaviors (22), which can negatively impact the mother’s health. In contrast to body dissatisfaction, women with a positive body image have demonstrated greater respect and care for their bodies, which may be associated with healthier eating behaviors and better food choices (23, 24).

In this sense, body appreciation stands out as an important construct to be studied in this population, as it is related to lower susceptibility to body dissatisfaction, the desire to change one’s appearance, the influence of external body ideals, and the development of disordered eating behavior (25). Among the various factors influencing the relationship with body image, self-compassion may play a relevant role amidst the challenges of motherhood for children with ASD. Increasing self-compassion can potentially neutralize the harmful effects of low body appreciation (26) by transforming negative emotions toward their bodies into a more positive emotional state, such as gratitude and appreciation for their physical form, as they adopt a more compassionate stance (27, 28). Furthermore, the influence of body image on eating behavior is evidenced by Ersoy et al., whose findings among men and women in Turkey indicate that emotional eating has an inverse relationship with body appreciation (19). Reinforcing this idea, it is observed that women are more likely to value their bodies and eat according to their hunger and satiety cues when they do not focus on how their appearance is perceived by others, demonstrating that avoiding self-comparison indirectly reduces emotional eating (29).

Furthermore, higher self-compassion scores were related to higher diet quality, body esteem, and intuitive eating scores, as well as lower emotional eating in a sample of mothers in Canada (30). Additionally, the association between self-compassion and emotional eating appears to be mediated by body esteem (31), justifying the development of studies that specifically address these themes in mothers of children diagnosed with ASD, in order to include them in the care network targeted at the treatment of this disorder. Based on this, it is hypothesized that high levels of self-compassion are directly associated with greater body appreciation, which, in turn, would be negatively correlated with emotional eating. Considering that caregiving overload can compromise body perception and foster dysfunctional eating behaviors, it is fundamental to investigate protective factors in this group. Therefore, the objective of this study was to evaluate the factors associated with body appreciation in mothers of children with ASD.

## 2 Materials and methods

This epidemiological, analytical, and cross-sectional investigation was carried out with women living in the state of Espírito Santo who are mothers of children living with ASD. Participants were chosen by “snowball” sampling, a non-probabilistic technique in which the selected individuals invite new participants from their network of friends and acquaintances (32). For this, the project of this research was publicized on the researchers’ social media, requesting the support of social media profiles that deal with ASD and entities and professionals related to its diagnosis and treatment. At the time of data collection, no information on the national prevalence of ASD in Brazil existed, which made it difficult to calculate the necessary number of mothers for evaluation.

However, a question was included in the last Brazilian Institute of Geography and Statistics census about the presence of residents with an ASD diagnosis. Its results, published in 2025, indicate that about 1.2% of the Brazilian population lives with autism (33). These national and regional data were unknown at the time of collection, which made it impossible to calculate a precise sample size. The technique above was chosen for this reason. Data were collected in the first half of 2023. The following inclusion criteria were chosen: age over 18 years, being a mother and the main caregiver of a child aged up to 12 years with a medical diagnosis of ASD, and residence in the state of Espírito Santo, Brazil. No exclusion criteria were considered in this research.

### 2.1 Participants

The target population of this research consisted of mothers of children who were aged up to 12 years who had a medical diagnosis of ASD. Data were collected by a Google Forms^®^ questionnaire.

### 2.2 Measures

#### 2.2.1 Sociodemographic

Data related to participants’ age (in complete years, then categorized into age groups); marital status (“married/lives with a partner,” “single/lives without a partner”); race/color (self-reported as Black, Brown, white, Yellow, or Indigenous, according to the Brazilian Institute of Geography and Statistics); education (“complete secondary education,” “complete tertiary education/graduate degree); income (“up to two minimum wages,” “from two to five minimum wages,” “over five minimum wages”) (34) and occupation (“No” or “Yes”) were collected.

#### 2.2.2 Lifestyle habits

Participants’ tobacco and alcohol consumption were assessed according to the Consensus Approach and Smoker Treatment (35). Participants who reported using tobacco, regardless of the amount or frequency, were considered smokers. For this study, the participants were categorized as “non-smokers,” “smokers,” and “former smokers.” Alcohol consumption was categorized according to participants’ self-reported information into “does not consume,” “consumes,” and “consumed, but stopped” (35). Participants were asked if they practiced any physical activity. Those who reported at least 30 min of continuous or accumulated moderate physical activity per day at least five days a week were classified as “physically active,” whereas those who failed to meet these standards were deemed and “physically inactive” (36).

#### 2.2.3 Anthropometric

The data on current weight (kilograms) and height (meters) were self-reported. Nutritional status was assessed by BMI (obtained by dividing weight by height squared) and classified according the World Health Organization criteria (36), which characterizes BMI below 18.5 as underweight; 18.5–24.99, as eutrophic; ≥25.0, as overweight; 25.0–29.99, as pre-obese; 30.0–34.99, as class I obesity; 35.0–39.99, class II obesity; and ≥40, as class III obesity. Numerical BMI data was used for the inferential analyses rather than the categories since the outcome is a continuous numerical data, thus enabling the evaluation of the coefficient of the relationship between these variables.

#### 2.2.4 Sociodemographic and clinical aspects of the child

Gender was classified as “female” and “male.” Children’ age was categorized into age groups. Information on the level of support the children required was classified: Level 1 “requiring support,” Level 2 “Requiring substantial support,” and Level 3 “Requiring very substantial support” (37).

#### 2.2.5 Emotional eating

To evaluate this construct, the questionnaire “Three Factor Eating Questionnaire” – 21-item version (TFEQR-21) was used. This self-administered instrument divides its 21 items into three subscales: cognitive dietary restraint, emotional eating, and lack of eating control. Only the emotional eating subscale—which includes six items associated with the predisposition to overeating as a response to negative emotions—was evaluated in this study. Its score ranges from 0 to 100; the higher the score, the more present the evaluated eating behavior (38).

#### 2.2.6 Self-compassion

The self-compassion scale, developed by Neff (2003) and validated for Brazil by Souza and Hutz (2016), consists of 26 items in six subscales, three of which are scored as positive (benevolence, common humanity, and mindfulness) and three of which are scored as negative (self-evaluation, isolation, and overidentification). Answer options follow a five-point Likert scale ranging from 1 (almost never) to 5 (almost always). The average score on each of the six subscales is then calculated (after reverse coding of negative items) to create a total self-compassion score (39).

#### 2.2.7 Body appreciation

This construct was evaluated by the “Body Appreciation Scale-2” developed by Tylka and Wood-Barcalow (69) and translated and validated for Brazil by Junqueira et al. (2019). For each item, the following response scale should be used: 1 = “never,” 2 = “rarely,” 3 = “sometimes,” 4 = “often,” 5 = “always.” The final score is represented by the average of items 1 to 10, and the higher this score, the higher the body appreciation (39).

### 2.3 Procedures

Initially, individuals who wished to participate in this research had to check the option “accepted” in the provided informed consent form after they were informed about the inclusion criteria for this research. Then, they answered the provided semi-structured online questionnaire.

**Table 1:** Sociodemographic, clinical, lifestyle, and behavioral data of mothers and children with ASD.

Variables	*N*	%
Age** (*n* = 141)	36.72 ± 6.71	
Age group (n = 142)		
20–29 years	26	18.3
30–39 years	67	47.2
>40 years	49	34.5
Marital status (n = 143)		
Living with a partner	116	80.1
Living without a partner	27	18.8
Race/color (n = 143)		
White	60	42
Black or Brown	83	58
Schooling (n = 143)		
Complete secondary education	49	34.3
Complete tertiary education/Graduate degree	94	65.7
Income (n = 136)		
Up to 2 minimum ages	50	36
From 2 to 5 minimum wages	44	31.7
More than 5 minimum wages	45	31.5
Occupation (n = 142)		
No	55	38.7
Yes	87	61.3
Drinking (n = 143)		
No	81	56.6
Yes	48	33.6
Have ingested but quit	14	9.8
Smoking (n = 143)		
No	126	88.1
Yes	12	8.4
Have smoked but quit	5	3.5
Physical activity (n = 143)		
No	106	74.1
Yes	37	25.9
Emotional eating score* (*n* = 139)	66.7 ± 50	
Self-compassion score* (*n* = 143)	2.8 ± 0.9	
Body appreciation score* (*n* = 136)	2.5 ± 1.5	
BMI* (*n* = 140)	28.0 ± 8.4	
Nutritional status (n = 143)		
Low weight/eutrophy	42	29.8
Overweight/obesity	99	70.2
Sex of the child (n = 142)		
Female	22	15.5
Male	120	84.5
Child’s age group (n = 143)		
0–5 years	89	62.2
≥6 years	54	37.8
Child’s level of support (n = 143)		
Level 1	64	44.8
Level 2	46	32.2
Level 3	25	17.5
I cannot opine	8	5.6

*Data shown as p50 ± the interquartile range (IQR); **Data shown as mean and standard deviation; ASD, autism spectrum disorder.

### 2.4 Statistical analysis

Based on the collected data, statistical analysis was performed on JASP 18.0 (*Jeffreys’s Amazing Statistics Program*). Variable normality was assessed by the Shapiro–Wilk test. Means (with standard deviation), medians (with interquartile range), and absolute and relative frequencies were used to describe the study variables. The Student’s *t*-test was used to analyze the differences between means and the Mann–Whitney test, to analyze medians. To analyze proportion differences, the chi-squared (X^2^) or Fisher’s exact tests were used. Spearman’s correlation analysis was used to evaluate the relationship between the numerical variables. The significance level for all tests was 5%. Multivariate analysis (multiple linear regression for the first outcome—body appreciation, and binary logistic regression for the second outcome—children’s food selectivity) was used to quantify the participation of the independent variables in the outcome of interest.

## 3 Results

Table 1 characterizes the 143 women who participated in this study. Most participants were aged from 30 to 39 years (47.2%) with 36.7 ± 6.7 years mean age. Most lived with a partner (*n* = 116, 80.1%), were Black or Brown (58%), had complete tertiary education/graduate degree (65.7%), worked (61.3%), and had a family income of up to two minimum wages (42.4%). Clinical data showed a predominance of overweight (overweight/obese) participants (70.2%), with a median BMI of 28 ± 8.4 kg/m^2^. Regarding lifestyle habits, most neither drank alcohol (56.6%) nor used tobacco (88.1%) and were sedentary (74.1%). The median body appreciation score equaled 2.5 points (minimum = 1.1; maximum = 4.9). Their children’s data show that most were boys (84.5%) who were aged from zero to five years (62.2%), needed level 1 support (44.8%), and had food selectivity (68.5%).

Table 2 shows the sociodemographic, clinical, and lifestyle data of mothers and children with ASD according to the total body appreciation score. This score was higher in those aged over 40 years (*p* = 0.043), with a higher education/graduate degree (*p* = 0.006), income from two to five minimum wages (*p* = 0.035), and an occupation (*p* = 0.020). Participants who were eutrophic (*p* < 0.001), who reported engaging in physical activity (*p* = 0.002), and whose children were aged over six years (*p* = 0.007) also had higher body appreciation.

**Table 2:** Sociodemographic, clinical, lifestyle, and behavioral data of mothers and children living with ASD, distributed according to body appreciation score.

Variables	Body appreciation score		
	Mean	SD or IQR	*p*-value
Age^1^	36.62	6.71	**0.029**
Age group^2^			**0.043**
20–29 years	2.35	1	
30–39 years	2.6	1	
>40 years	2.94	1	
Marital status			0.889
Living with a partner	2.67	0.96	
Living without a partner	2.64	1.15	
Race/color			0.576
White	2.77	1.04	
Black or Brown	2.63	0.96	
Schooling			**0.006**
Complete secondary education	2.35	0.97	
Complete tertiary education/graduate degree	2.84	0.97	
Income^2^			**0.035**
Up to 2 minimum ages	2.36	0.99	
From 2 to 5 minimum wages	2.89	0.98	
More than 5 minimum wages	2.7	0.93	
Occupation			**0.02**
No	2.41	0.99	
Yes	2.82	0.97	
Drinking^2^			0.428
No	2.57	0.94	
Yes	2.78	1.04	
Have ingested but quit	2.84	1.15	
Smoking^2^			0.746
No	2.69	1	
Yes	2.48	1.06	
Have smoked but quit	2.52	0.86	
Physical activity			**0.002**
No	2.51	0.95	
Yes	3.11	0.99	
BMI^2^*	28	8.4	**<0.001**
Emotional eating score^3^*	66.7	50	**<0.001**
Self-compassion score^3^*	2.8	0.9	**<0.001**
Nutritional status			**<0.001**
Low weight/eutrophy	3.15	1.03	
Overweight/obesity	2.44	0.9	
Sex of the child^3*^			
Female	2.7	2.1	
Male	2.4	1.4	
Child’s age group			**0.007**
0–5 years	2.5	0.91	
≥6 years	2.97	1.07	
Child’s level of support^2^			0.431
Level 1	2.63	0.99	
Level 2	2.75	1.08	
Level 3	2.78	0.95	
I cannot opine	2.16	0.56	

Student’s t-test; ^1^Spearman’s correlation; ^2^ANOVA; ^3^ Mann–Whitney test; SD, standard deviation; BMI, body mass index; ASD, autism spectrum disorder; In bold, statistically significant values (*p* < 0.05).

*Data shown as p50 ± the interquartile range (IQR).

Figure 1 shows Spearman’s correlation analysis, which found a positive, weak, and significant correlation between body appreciation, mother’s age (R = 0.188; *p* = 0.029), and child’s age (R = 0.204; *p* < 0.05) and a strong and significant positive correlation between self-compassion and the outcome (R = 0.520; *p* < 0.001). It also found a negative, moderate, and significant correlation between body appreciation and BMI (R = −0.451; *p* < 0.001) and a strong and significant negative correlation with emotional eating and the outcome variable (R = −0.563; *p* < 0.001).

**Figure 1:**
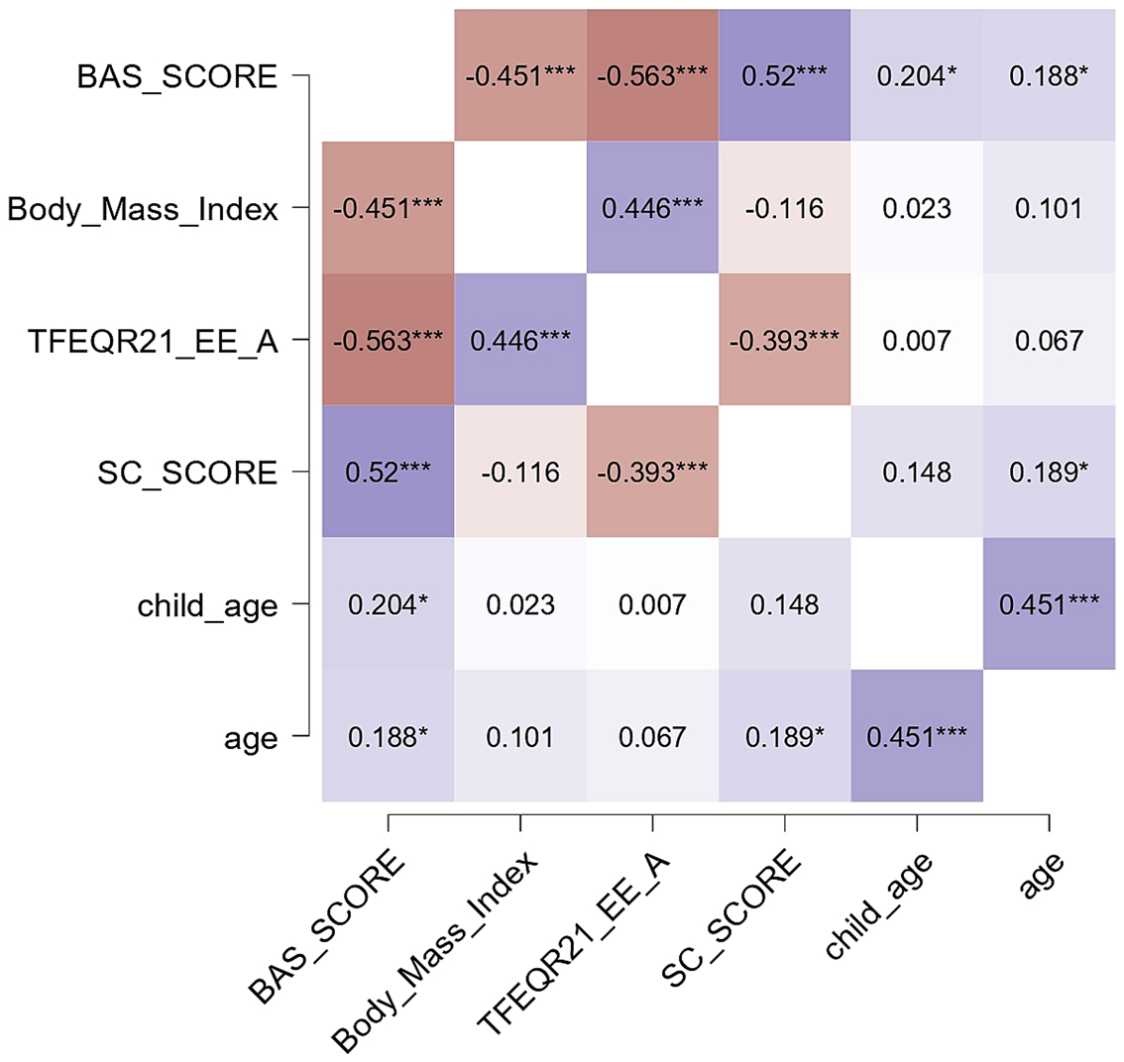
Spearman’s correlation heatmap between body appreciation score, BMI, emotional eating, self-compassion score, child’s age, and mother’s age. BAS score, Body Appreciation Scale score; BMI, body mass index; TFEQR21 EE, three-factor eating questionnaire - R21 - emotional eating; SC score, self-compassion score; Child age: Child’s age; Age, mother’s age. *p < 0.05; **p < 0.01; *p* < 0.001.

The multiple linear regression analysis (Table 3) showed that physical activity (*β* = 0.293; *p* = 0.044) and children’s age above six years (*β* = 0.329; *p* = 0.016) predicted a higher score on the scale that assesses body appreciation. Each one-point increase in BMI decreased the body appreciation score by 0.034 points (*β* = −0.034; *p* = 0.005). Likewise, each one-point increase in the emotional eating score decreased the body appreciation score by 0.010 points (*β* = −0.010; *p* < 0.001). A one-point increase in the self-compassion score raised body appreciation scores by 0.440 points (*β* = 0.440; *p* < 0.001), showing the positive relationship between these constructs.

**Table 3:** Multiple analysis of the body appreciation score of mothers of children diagnosed with ASD.

Variables	Unstandardized coefficient (95% CI)	Standardized coefficient	*t*	*p-*value
Emotional eating score	-0,010 (−0.015, −0.005)	−0.323	−4.154	**<0.005**
Self-compassion score	0,440 (0.253, 0.627)	−0.210	4,666	**<0.001**
BMI	−0,034 (−0.057, −0.011)			**0.005**
Physical activity			−2.879	
No	1			
Yes	0,293 (0.008, 0.577)			**0.044**
Schooling			1.845	
Complete secondary education	1			
Tertiary education/ Graduate degree	−0,070 (−0.404, 0.264)	0.324	0.661	0.680
Income				
Up to 2 minimum wages	1			
From 2 to 5 minimum wages	0,177 (−0.183, 0.536)		0.974	0.332
More than 5 minimum wages	0,058 (−0.317, 0.432)		0.305	0.761
Child’s age group				
0–5 years	1			
≥6 years	0,329 (0.064, 0.594)		0.016	**0.016**
Age group				
20–29 years	1			
30–39 years	0,114 (−0.246, 0.474)		0.628	0.531
>40 years	0,202 (−0.230, 0.634)		0.925	0.357

In bold: statistically significant values (*p* < 0.05); BMI, body mass index. CI, confidence interval; ASD, autism spectrum disorder; R^2^ = 0.554; Adjusted R^2^ = 0.515; *F*-value = 14.15; Degrees of freedom (df) = 10 and 114.

Thus, physical activity, the child’s age exceeding six years, and greater self-compassionate behavior directly predicted higher body appreciation. On the other hand, a higher BMI and emotional eating score predicted lower body appreciation in the studied sample.

## 4 Discussion

In the present study, we tested a hypothesized model in which mothers of children with ASD who exhibit more self-compassionate behavior show greater body appreciation, whereas those who engage in more emotional eating demonstrate lower scores in this construct. The results supported our hypotheses: higher levels of self-compassion were associated with higher body appreciation scores, while emotional eating was negatively associated with this construct. This finding expands existing research on the motherhood of children living with ASD, as current literature lacks studies addressing these concepts specifically in this population. Although body appreciation has been associated with positive behaviors in mothers (40), to the authors’ knowledge, no studies have evaluated this construct in mothers of children living with ASD. Our findings indicate that mothers with higher levels of self-compassion have a more positive perception of their own bodies. Although the relationship between self-compassion and body appreciation has already been observed in convenience samples, such as college students (41), it is fundamental to highlight that, in the context of ASD motherhood, this mechanism assumes a distinct adaptive function. For these caregivers, self-compassion may act as a psychological resource that allows for the decoupling of personal worth from aesthetic standards—a vital mechanism that focuses on body functionality as a support for the daily demands of caregiving (18).

Another study demonstrated the effectiveness of self-compassion in restoring body appreciation after appearance-related ruminations in female adolescents (42). Also in line with our findings, a pilot study evaluated the effect of incorporating self-compassion meditation to improve body image in 71 women during pregnancy and postpartum, where the intervention group reported improved body appreciation compared to the control group (43). This relationship may occur because self-compassionate parents are less likely to be self-critical or to have negative thoughts and feelings; instead, they experience self-acceptance and hope for the future (3), which may be related to greater body appreciation. The study by Cox et al. highlights the relevance of self-compassion for body appreciation by identifying that higher levels of this construct on a given day are associated with greater valuing of one’s own body, as well as more positive and fewer negative attitudes toward oneself (44). The authors suggest that encouraging and teaching individuals to treat themselves with more self-compassion than usual can help them develop a more positive relationship with their bodies, in addition to improving their relationship with food (45). A study with 147 Brazilian mothers of children with ASD revealed that a 0.280-point increase in the self-compassion score resulted in a one-point increase in the body appreciation score (46), demonstrating the importance of this construct as a protective factor for the physical and psychological well-being of this population.

A positive correlation was found between body appreciation and both the mother’s age and the child’s age. Specifically, the body appreciation score was higher in women over 40 years of age, and having a child older than six years was positively associated with greater body appreciation. This finding suggests a possible maternal adaptation over time, indicating that as age increases, levels of body appreciation also rise (47). Similarly, a study of 541 women demonstrated a positive correlation between age and body appreciation (48), which may be related to the child’s older age. Some authors theorize that this scenario is justified by older women distancing themselves from social ideals, as they accept and appreciate their bodies for their functionality and health to the detriment of physical appearance (49). Furthermore, the positive relationship between age and body image may be related to the fact that many other aspects of women’s bodies and lives become more important than appearance as they age (50). In the specific context of ASD, this maturation of body perception may be conditioned by the stabilization of therapeutic routines and the reduction of acute overload—factors frequently observed in the years following the initial diagnosis (51). Over time, mothers’ perceptions move beyond initial difficulties toward the possibility of their children leading healthy lives (52), which can significantly decrease stress levels.

In our regression model, physical activity emerged as a factor independently associated with body appreciation, a phenomenon also observed in studies involving other populations (44, 53–56). In the context of motherhood, qualitative evidence suggests that mothers who exercised because they appreciated their own bodies felt less pressure to engage in rigorous exercise, as their goals were unrelated to maintaining appearance (40). Although there is a gap in studies addressing the relationship between body appreciation and physical activity specifically among mothers of children with ASD, the findings of the present study contribute to the existing literature, which correlates physical activity with greater body appreciation, suggesting that regular exercise aids in the appreciation of physical capabilities and body functionality (57). However, it is essential to emphasize the importance of a support network that enables these mothers to dedicate time to self-care.

BMI is inversely related to body appreciation in the studied sample, corroborating the differences observed across nutritional status categories. This finding converges with a study of 531 Korean adult women aged 20–77, which identified the same relationship (48). According to a meta-analysis, the negative association between body appreciation and BMI is significantly more pronounced among women (58); in this group, the higher the Body Mass Index, the lower the acceptance of one’s own body (59). Added to this, the incessant cultural pressure regarding female appearance highlights women as a risk group for body image disorders (47, 60). The media portrays thin bodies as exclusively desirable and fosters discomfort with one’s own body, generating distress and anxiety. In this sense, self-perception is impaired as it stems from a stigmatized identity and social comparisons, which can negatively affect body appreciation for those whose bodies are further from the standard (58). This relationship becomes more complex in the scenario of ASD motherhood, as participation in leisure activities and self-care is even more challenging (61). Furthermore, a study of 313 older Chinese men and women points to body dissatisfaction and body image inflexibility as significant mediators of the relationship between BMI and body appreciation in women, indicating that this relationship is not restricted to adolescent girls or young women (62). Finally, excess weight may restrict the choice of physical activities, a factor that helps explain why a high BMI frequently reduces body appreciation (58).

Additionally, it was found that emotional eating is inversely associated with body appreciation (63). This finding is extremely relevant considering that mothers of children with ASD experience high levels of stress, which is positively associated with emotional eating, specifically among mothers of autistic children (8). A recent study with parents of children with ASD found that total stress scores were associated with emotional eating, primarily in mothers (64). Research involving 573 women aged 38 to 50, using the same instrument as the present study, demonstrated a positive association between emotional eating and BMI (65). This suggests that a dysfunctional relationship with food can foster weight gain, which in turn is related to lower body appreciation. This association has been observed in various studies, generally with younger population groups (58, 62, 66, 67), reaffirming the need to study this concept within the population of mothers of children with ASD.

A study involving 288 adults and 18 older adults from the United States divided the sample into three behavioral profiles, finding that the profile with the lowest body appreciation presented the lowest adaptive eating behaviors and the highest levels of uncontrolled and emotional eating. Furthermore, it evidenced that the BMI of those who engaged in more emotional eating was higher, and the significant negative correlation found between this indicator and body appreciation scores may trigger emotional eating (68). In the context of ASD motherhood, a study of 147 Brazilian mothers of autistic children revealed that a 0.209 kg/m^2^ increase in BMI raised the emotional eating score by one point (46), highlighting the influence of nutritional status on this population’s eating behavior. Additionally, the intense level of stress experienced by these mothers must be emphasized, as it may explain the relationship between emotional eating and body appreciation (30).

The present study provides important information that can assist in mitigating health damages among mothers of children with ASD, as well as in developing strategies for health and well-being promotion and their inclusion in the care network directed at the disorder. The relevance of this study is also highlighted by providing innovative data concerning this population, which is currently under-investigated. However, the study has some limitations. Data were collected through an online questionnaire, and weight and height measurements were self-reported. Nevertheless, this research format expands access to mothers who face difficulties in traveling due to being in constant care of their children, allowing them to be part of the study.

Furthermore, data collection through virtual means simplifies fieldwork, in addition to being low-cost and enabling greater participation (51). The use of the “snowball” sampling technique through social media constitutes a methodological limitation regarding selection bias, preventing the extrapolation of findings. Since recruitment depended on referrals and participation in ASD-related groups, the sample tends toward homogeneity in terms of sociodemographic and psychosocial characteristics. Consequently, mothers in conditions of greater isolation or digital vulnerability may have been underrepresented, requiring caution in the generalization of the results. Another limiting point of the study is its cross-sectional design, which precludes the determination of temporal relationships between the studied variables. Finally, it should be noted that questionnaire responses were self-reported, which allows for the potential underestimation or overestimation of data.

## 5 Conclusion

This cross-sectional study identified that mothers of children with ASD who exhibited higher self-compassion scores, engaged in physical activity, and had children over six years of age demonstrated greater body appreciation. In contrast, higher levels of emotional eating and a higher BMI were associated with lower scores in this construct. These results suggest that self-compassion may act as a protective factor for a more positive relationship with the body, whereas emotional eating may represent a risk factor for negative body image. From a practical perspective, these findings highlight the relevance of interventions that promote the development of self-compassion, particularly in support programs targeted at mothers of children with ASD, considering the specific challenges faced by this population and their impact on body perception and eating patterns. Such initiatives can contribute to the promotion of comprehensive health for this group by integrating preventive and management approaches in mental health. However, further studies with mothers of children with ASD are necessary to establish solid foundations for both theories and practices focused on body image and eating behavior within the context of this population.

## Data Availability

The datasets/Supplementary material for this article can be found online at: https://figshare.com/s/648ead329fd1c0224383.
